# Influence of care group participation on infant and young child feeding, dietary diversity, WASH behaviours and nutrition outcomes in rural Zimbabwe

**DOI:** 10.1136/bmjnph-2023-000627

**Published:** 2023-09-14

**Authors:** Tonderayi Mathew Matsungo, Faith Kamazizwa, Tafadzwa Mavhudzi, Starlet Makota, Blessing Kamunda, Calvin Matsinde, Dexter Chagwena, Kudzai Mukudoka, Prosper Chopera

**Affiliations:** 1 Department of Nutrition, Dietetics and Food Sciences, University of Zimbabwe, Harare, Zimbabwe; 2 Nutrition Action Zimbabwe (NAZ), Harare, Zimbabwe; 3 UNICEF Zimbabwe, Belgravia, Zimbabwe

**Keywords:** Malnutrition, Nutrition assessment, Preventive counselling, Nutrient deficiencies, Nutritional treatment

## Abstract

**Background:**

The care group approach (CGA) is a community-based nutrition behaviour change strategy centred on ‘peer-to-peer learning’ through women support groups.

**Objective:**

To assess the impact of the CGA on the adoption of appropriate infant and young child feeding (IYCF), dietary diversity and water, sanitation and hygiene (WASH) practices, and associated nutrition-related outcomes.

**Methods:**

A retrospective cohort study used a mixed-method approach in selected rural districts in Zimbabwe in June 2022. A structured questionnaire was used to collect data on IYCF, diet quality, WASH and child morbidity. Binary logistic regression was used to evaluate the association between exposure and outcome. Significance was at p<0.05.

**Results:**

A total of 127 exposed and 234 controls were enrolled. There was no significant difference between exposed and controls on the prevalence of; diarrhoea (p*=*0.659), cough (p=0.191) and fever (p=0.916). No significant difference was observed in the proportion ever breastfed (p=0.609), Children with Adequate Dietary Diversity Score (p=0.606) across the two groups. However, the proportion of families with adequate Household Dietary Diversity Score (HDDS) (p=0.005) and Minimum Dietary Diversity for Women (MDD-W) (p=0.009) were significantly higher in exposed than controls. Knowledge on all promoted behaviours was significantly higher in the exposed than in controls with the exception of exclusive breast feeding. While the practices were significantly higher in exposed compared with controls for: ‘Appropriate complementary feeding for children aged 6–24 months’ (p=0.001), ‘good nutrition for women of reproductive age’ (p=0.001), ‘production and consumption of diverse nutritious food’ (p=0.001) and ‘production and consumption of biofortified crops’ (p=0.001).

**Conclusions:**

The results showed that CGA potential to increase knowledge and achieve nutrition and health-related behaviour change in low-income settings if integrated into existing community programmes. Interestingly, HDDS and MDD-W were significantly higher in exposed than controls. However, more research is required to obtain conclusive results.

**WHAT IS ALREADY KNOWN ON THIS TOPIC**

- There is limited evidence showing the potential of care group approach towards improved infant and young child feeding (IYCF), dietary diversity, water, sanitation and hygiene (WASH) behaviours and practices in low-income settings.

**WHAT THIS STUDY ADDS**

- These findings add on to existing evidence demonstrate that shows the potential benefits of using the care group approach as a vehicle to integrate nutrition within existing rural agriculture and livelihoods programmes to promote adoption of diet diversity, IYCF and WASH-related behaviours.

**HOW THIS STUDY MIGHT AFFECT RESEARCH, PRACTICE OR POLICY**

- There is a need to use lessons implementing an integrated community resilience building programme (ZRBF) to promote linkages between agriculture, nutrition and health sectors. The multisectoral approach is required to ensure sustainability and possible scaling up to non-participating districts.

## Introduction

The burden of malnutrition has remained a public health concern for Zimbabwe. Prevalence of stunting is still high at 29.4%.^1^ Major malnutrition drivers in Zimbabwe have been intensified by economic, social, environmental and political shocks and stresses, many of which have devastating effects on vulnerable populations.^2^ When faced with challenges, communities often cope by striving towards food quantity as opposed to food quality, thereby compromising their nutrition well-being.^3^ Stunting is the leading form of malnutrition among children younger than 5 years worldwide^4^ In Zimbabwe, one in three children under 5 years is affected by stunting, 29.4%.^1 5^ The diets in Zimbabwe are predominantly cereal based with limited consumption of fruits, vegetables and animal source foods thus lacking in key micronutrients.^2^ Only 42% of infants (under 6 months) are exclusively breastfed, with only 10% children (6–23 months) receiving a minimum acceptable diet.^2^ Unfortunately, in Zimbabwe, improving diet quality and reducing the prevalence of stunting has been a challenge.^6^ Therefore, there is need to focus interventions that use a multisectorial approach to effectively reduce stunting by tackling the underlying determinants.

The care group approach (CGA) is often fronted by UN agencies and international NGOs to drive improved child nutrition in many low-income countries and characterised by training of community health workers and neighbour women groups (NWGs) hinged on concept of peer to peer support for the adoption of appropriate nutrition and health-related practices.^7^ The CGA has strengthened communities’ resilience to repeated shocks.^8^ The limited available evidence in Zimbabwe show that CGS improved infant and young child feeding (IYCF), water, sanitation and hygiene (WASH) behaviours and practices and has opportunity to include various age groups such as adolescents.^9–12^ The results agree with findings from Kenya,^13^ Mozambique,^14^ as well as other settings.^15^

The Zimbabwe Resilience Building Fund (ZRBF) project was piloted in 18 districts to mainstream nutrition via the CGA to promote social behaviour change. The aim was to integrate nutrition into existing resilience and community health structures. Therefore, this study investigated the impact of the CGA on the adoption of appropriate IYCF, dietary diversity and WASH practices and associated nutrition-related outcomes.

## Methods

### Study setting

**Figure 1:**
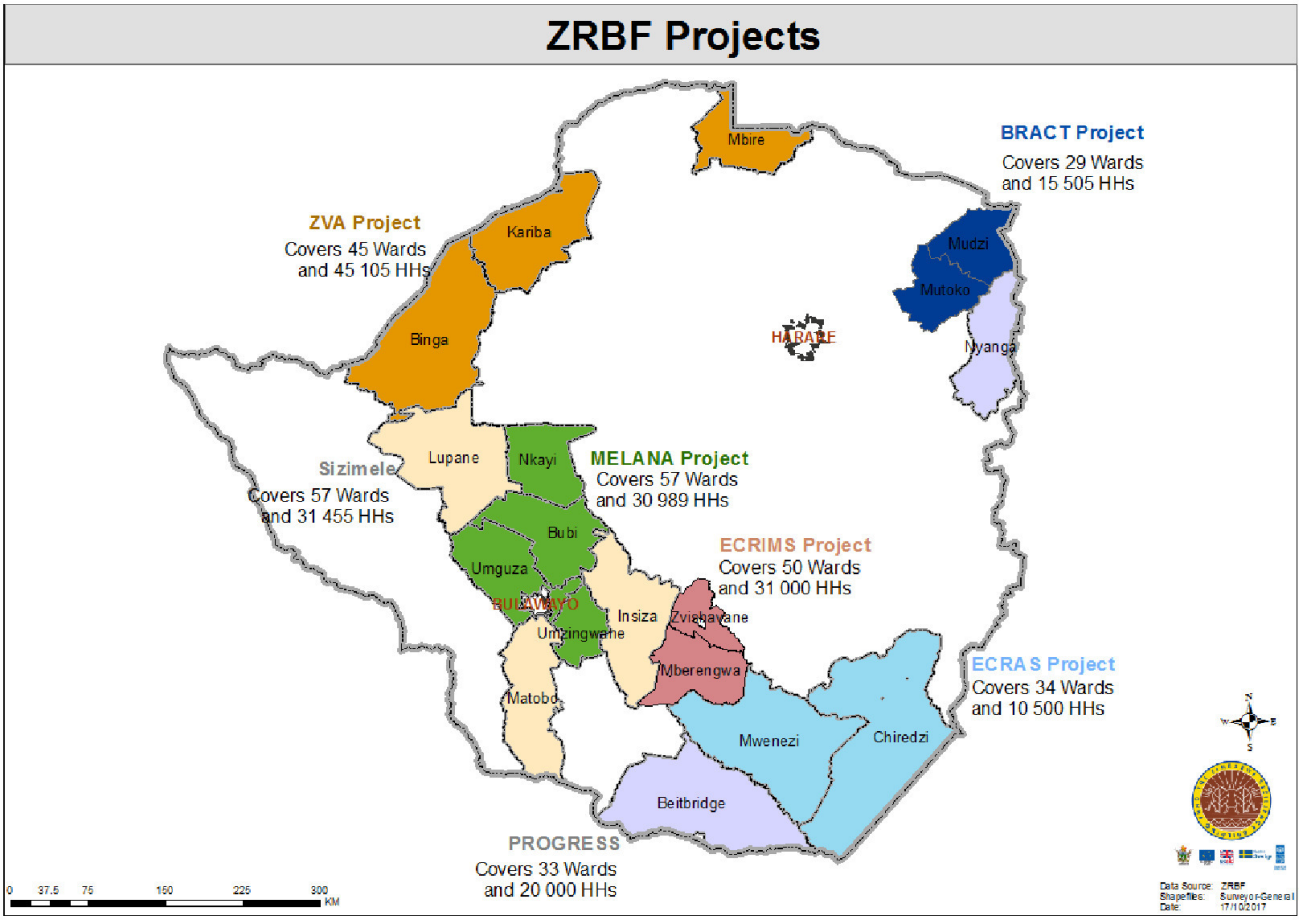
Map showing the ZRBF project implementation districts in Zimbabwe. HH, household head; ZRBF, Zimbabwe Resilience Building Fund.

This study was conducted in seven randomly selected districts participating in the ZRBF programme in Chiredzi (Masvingo), Beitbridge (Matabeleland North), Mutoko (Mashonaland East), Kariba (Mashonaland West), Mberengwa (Midlands), Bubi (Matabeleland North) and Insiza (Matabeleland South) (figure 1). These districts are in agro-ecological regions 4 and 5 and were participating in the nutrition sensitive agriculture and resilience building programme (ZRBF) and in addition the beneficiaries also participated in the nutrition focused SBCC care groups.

### Study design, subjects and sampling

The study used an unmatched retrospective cohort design. Zimbabwe has 60 rural districts, and the lower-level administrative unit is the ward. Wards contain several villages and each village consists of approximately 80–100 households. In this study, our sampling unit was the household. Seven districts were randomly selected out of the possible 18 pilot districts (exposed) implementing the ZRBF programme. The control households were also obtained from the same province and district but in wards that were not implementing ZRBF activities. The questionnaire was targeting women of reproductive age (15–49) from the sampled households. We used a ratio of exposed to controls of 1:2, respectively, according to by Sullivan and Soe.^16^ The sample size calculation was based on previous methods by Fleiss^17^ at 95% CI and 80% power, a 1:2 exposed to controls ratio, to detect an OR of 2.0 or greater. The calculated sample size was 411 households (137 exposed and 274 controls). This was and distributed across the seven sampled districts.

### The care group approach

In ZRBF, the CGA was constructed based on global guidance, where volunteers (lead mothers) motivate mothers (neighbour women) to adopt promoted nutrition and health behaviours.^7^ The lead mothers have a manageable workload of up to 15 neighbour women constituting a care group, where they meet at least once a month for support and supervision. The detailed description of CGA model in the Zimbabwean context has been described previously by Macheka *et al*. The ZRBF care groups promoted seven behaviours: (1) exclusive breast feeding (EBF) for children from birth to 6 months; (2) offer children aged 6–23 months, timely, adequate and diverse complementary feeding with continued breastfeeding up to 2 years and beyond (at least four of the eight food groups); (3) consumption of diverse foods by women of childbearing age (15–49 years); (4) mothers of children 0–59 months wash their hands at the five critical times; (5) food preservation of at least four types of foods from the recent farming season for household consumption later in the year; (6) household production and consumption of diverse nutritious foods including neglected underused foods (eg, small grains and wildly harvested vegetables) at least three times per week) and (7) purchase of nutritious, diverse foods including animal source foods and legumes using agricultural income.

### Data collection and tools

The study used a mixed-methods approach was both quantitative (structured questionnaire) and qualitative data (focused group discussion (FGD) and key informant interview (KIIs)). The district teams of consisted of district a nutritionist (supervisor) and enumerators (nutrition assistant, environmental health officer, agriculture extension officer). The data collection was coordinated by team of national supervisors from the University of Zimbabwe. The use of enumerators who reside in the communities ensured context specific interviewing for the structured questionnaire ‘prolonged involvement approach’.^18^

#### FGDs and KIIs

Qualitative data were collected for the purposes of triangulation using FGD and KII guides. The KII targeted district and ward-level government and partner staff (district medical officer, village health workers, lead mothers). The questions focused on KII’s perceptions on the impact of CGA on: diversified crop and livestock production, household consumption patterns, opportunities and barriers to adoption of promoted behaviours, sustainability of the intervention and recommendations.

While, FGDs were only conducted with women of reproductive age (15–49 years) with at least 1–2 FGD per district. The FGD questions addressed the following themes: functionality, relevance, awareness, perceived barriers and facilitators to adoption of promoted behaviours, family and community support systems, stories of change, recommendations and sustainability of the groups.

#### Structured interviewer-administered questionnaire

A validated questionnaire adapted from the Zimbabwe Vulnerability Assessment Committee survey.^2^ The questionnaire had the following sections: demographics, socioeconomic information, participation in care groups, agricultural, WASH, IYCF practices. The questionnaire assessed the seven ZRBF promoted behaviours in a series of yes or no questions. The questionnaire was converted into an electronic version and uploaded on to android tablets using the Kobo Collect Toolbox application (Kobo, Cambridge, UK). The previously validated android-based questionnaire was administered in local language by trained enumerators who used Samsung A7 tablets. The enumerators were trained for a week before the survey. The training covered study background, protocol, fieldwork logistics and role plays using the data collection tools. The following previously validated questionnaire modules that assessed household, women and child dietary diversity adopted:

#### Household Dietary Diversity Score

Household diet diversity is used to measure the quality of family diets, especially macronutrients and micronutrients. Food intake data were collected using the 24-hour recall method. This consisted of accurately recalling, describing and/or computing food and drink consumed in her 24 hours or the day before the interview.^19^ The latter mentioned 12 food groups were used to calculate Household Dietary Diversity Scores (HDDS): (1) cereals, (2) roots and tubers, (3) vegetables, (4) fruits, (5) meat, poultry and offal, (6) eggs, (7) fish and seafood, (8) pulse, legumes and nuts, (9) milk and milk products, (10) oil/fats, (11) sugar/honey and (12) miscellaneous. Households that have consumed one of the above-mentioned food groups within the last 24 hours receive a score of 1 vs a score of 0 if the food was not consumed. The HDDS variable is calculated per household. The range of values for this variable ranged from 0 to 12.^20^

#### Minimum Dietary Diversity for Women

Dietary diversity thresholds for women were measured according to the FAO guidelines for measuring dietary diversity thresholds for women.^21^ This indicator measures the adequacy of micronutrients in women’s diets at the population level. Women of childbearing age (15–49 years) recorded all food consumed during the previous day and night (last 24 hours) at home or on the go. Foods were assigned to 10 recommended food groups: (1) grains, roots and tubers, (2) pulses, (3) nuts and seeds, (4) dairy, (5) meat, poultry and fish, (6) eggs (7), dark leafy greens and vegetables, (8) other vitamin A-rich fruits and vegetables, (9) other vegetables and (10) other fruits. The threshold for adequacy was defined as five or more food groups.

#### Child Dietary Diversity Score

Minimum dietary diversity (MDD) child was determined according to the new IYCF indicator guidelines.^22^ Breastmilk was included as the eighth food group and the cut-off for adequacy was at least five out of eight food groups, including breastmilk. The food groups included therefore were: breastmilk, grains roots and tubers, legumes and nuts, dairy products, flesh foods, eggs, vitamin A rich fruits and vegetables, and other fruits and vegetables.

#### Child morbidity

Prevalence of the three most common childhood ailments, diarrhoea, cough and fever in the study children was assessed by research assistants by interviewing the caregivers . The recall period for all symptoms was 2 weeks. Prevalence was determined as number of reported events over total number of recalls.

### Qualitative data collection

#### Focus group discussions

The FGDs consisted of 6–12 people, 1 per district (7 FGDs). Women of reproductive age (15–49 years) and beneficiaries of the care groups were randomly selected to participate in FGDs by the enumerators. FGD sites were conveniently selected by the enumerators. A focus group guide was developed specifically for the study. The FGDs were held up to the point where consecutive discussions revealed no additional data (saturation).

### Key informant interviews

A total of 37 key informants were purposively selected and they included government officials (Agritex officers, district nutritionists, nutrition assistants, sisters in charge and village health workers), programme officers (ZRBF, NAZ) and community leaders (village heads), lead farmers and lead mothers.

### Data analysis

All quantitative data were exported to SPSS V.24 (IBM) from Kobo Collect data base. The frequency of each measured continuous outcome in each of the two groups (exposed and controls) was calculated. Retrospective cohort studies typically produce the OR to measure strength of association between the intervention (exposure) and outcome. Variables with p value greater than 0.1 (p>0.1) were excluded from the multivariate binary logistic regression model (by enter method) to find adjusted OR. Statistical significance was set at p<0.05. Qualitative data were analysed in Excel using content analysis to establish emerging themes. The most widely adopted six-step framework for conducting thematic analysis was employed. This consists of (1) researcher familiarisation with the data, (2) generating initial codes, (3) searching for themes, (4) reviewing themes, (5) defining and naming themes and (6) producing the report.^23^

## Results

### Sociodemographics

The study successfully enrolled 127 exposed and 234 controls from seven rural districts in Zimbabwe. Majority of the household heads were in the age group 25–64 years, exposed (75%) and control (64%) correspondingly (table 1). The similar trend was also observed for interviewee WRA (Women of Reproductive age). While the median and IQR age for household heads and interviewee (WRA) were 40 (18–62) and 50.5 (27.5–73.5), respectively. Factoring in care group participation the median (IQR) among WRA for exposed group and control group were 37 (19–55) and 40 (16–64) respectively. The household heads were mostly males, 79.3% in exposed and 72% in controls and most of these were married (exposed, 84.8% and 72.3% controls). Farming was the major economic activity in the study districts (exposed, 79.3% and 64.6% controls). While the most frequent level of education was primary level in exposed (45.7%) and secondary education in controls (45.7%).

**Table 1:** Sociodemographic characteristics of study households

Variable	Exposed n (%)	Control n (%)	P value*
Age of household head (HH)			
<24 years (youth)	8 (8.7)	49 (14.9)	0.124
25–64 years (adults)	69 (75)	210 (64)	
>65 years (seniors)	5 (5.4)	28 (8.5)	
Age of interviewee (WRA)			
<24 years (youth)	8 (9.1)	49 (15.6)	0.133
25–64 years (adults)	76 (86.4)	240 (76.4)	
>65 years (seniors)	4 (4.5)	25 (8.0)	
Sex of HH			
Male	73 (79.3)	236 (72)	<0.001†
Female	19 (20.7)	92 (28)	
Marital status of HH			
Divorced/separated	2 (2.2)	21 (6.4)	<0.001†
Married	78 (84.8)	237 (72.3)	
Never married	1 (1.1)	1 (0.3)	
Single	3 (3.3)	10 (3.0)	
Widowed	8 (8.7)	59 (18)	
Type of family composition			
Child headed	0 (0)	9 (2.7)	<0.001†
Extended family	10 (10.9)	82 (25)	
Nuclear	77 (83.7)	225 (68.6)	
Polygamous	5 (5.4)	12 (3.7)	
Income			
Casual labour	3 (3.3)	53 (16.2)	<0.001†
Employed	4 (4.3)	38 (11.6)	
Farmer	73 (79.3)	212 (64.6)	
Gold panning	6 (6.5)	5 (1.5)	
Vending	2 (2.2)	8 (2.4)	
Other	4 (4.3)	12 (3.6)	
Highest education level of head			
None	3 (3.3)	45 (13.7)	<0.001†
Primary	42 (45.7)	122 (37.2)	
Secondary	46 (50)	150 (45.7)	
Tertiary	1 (1.1)	11 (3.4)	
Size of family (median, IQR)	5 (4–7)	6 (4–7)	0.383

*Pearson’s χ^2^ test used except for size of family whereby Independent samples median test was used.

†P value significant at p<0.05.

WRA, Women of Reproductive age (15-49 years old).

### Child indicators of diet quality and morbidity

The results (table 2) showed both positive and negative trends. Although not statistically significant we observed lower prevalence of diarrhoea (p=0.659), cough (p=0.191) and fever (p=0.916), in the exposed group. While the negative trends included, lower proportion of ever breastfed in the exposed group (p=0.609), and lower proportion of Children with Adequate Dietary Diversity Score (CDDS) in the exposed group (p=0.606), an indication of poorer diet quality in exposed than controls. However, these trends were all not significant.

**Table 2:** Child indicators of diet quality and disease prevalence

Variable	Exposed n (%)	Controls n (%)	OR*	95% CI	P value*
Select IYCF indicators					
Ever breastfed (yes)	35 (97.2)	58 (95.1)	1.81	0.18 to 18.09	0.609
(No)	1 (2.8)	3 (4.9)			
Child Dietary Diversity Score					
<5 food groups†	26 (72.3)	41 (66.2)	0.79	0.32 to 1.95	0.606
≥5 food groups	10 (27.7)	21 (33.8)			
Disease prevalence					
Diarrhoea (yes)	8 (22.2)	16 (26.2)	0.80	0.30 to 2.12	0.659
(No)	28 (77.8)	45 (73.8)			
Cough (yes)	26 (72)	36 (59.1)	1.81	0.74 to 4.40	0.191
(No)	10 (28)	25 (40.9)			
Fever (yes)	8 (22.3)	13 (21.4)	1.06	0.39 to 2.86	0.916
(No)	28 (77.7)	48 (78.6)			

*Binary logistic regression OR.

†New eight food groups for children calculation was used (breastmilk is the new standalone group).

### Perceptions of the promoted behaviours

Based on the qualitative data (FGDs and KIIs), participants think that it is necessary to adopt the seven promoted behaviours so as to have well-nourished children and families and to maintain cleanliness at all times. The participants also appreciated the different livelihoods-based activities that were being promoted, the increased household income has positive impact on positive nutrition behaviours. Overall the exposed reported that participating in care groups resulted in less children getting sick, diarrhoea incidents and improved IYCF practices. This is linked to improved access to safe drinking water, having toilets and practice of overall hygiene and adoption of nutrition gardens and rearing of small livestock. Although, direct statistics are difficulty to find, our observations are that child diets have become more diversified and nutritious driven by the promoted concept of four-star diet (food groups).

### Household and adult indicators wash, diet quality, promoted behaviours

The results in table 3 revealed that knowledge and practice of all promoted behaviours except EBF were significantly higher in the exposed. Practice was significantly higher in exposed compared with controls for: ‘appropriate complementary feeding for children aged 6–24 months’ (p=0.001), ‘good nutrition for women of childbearing age’ (p=0.001), ‘production and consumption of diverse nutritious food’ (p=0.001) and ‘production and consumption of biofortified crops’ (p=0.001). These are positive findings are implying the possible impact of the CGA in promoting nutrition and health-related behaviours. However, the specific results of these indicators are presented in next section.

**Table 3:** Household and adult indicators WASH, diet quality, promoted behaviours

Variable	Exposed		Controls		OR*	95% CI	P value*
	n	%	n	%			
Dietary diversity							
HDDS (mean±SD)	6.4±2.5		5.6±2.4				0.005†‡
MDD-W<5 Food groups	26	28.3	142	43.3	1.94	1.17 to 3.21	0.009†
≥5 food groups	66	71.7	186	56.7			
Knowledge of promoted behaviours							
Listed at least ≥5 behaviours promoted	83	96.5	164	49.4	28.3	8.78 to 91.5	<0.001†
Listed <5 behaviours promoted	3	3.5	168	50.6			
Practice of the promoted behaviours							
Safe household food processing, preservation and storage (yes)	81	94.2	259	78	4.57	1.78 to 11.69	0.002†
(No)	5	5.8	73	22			
Exclusive breastfeeding (yes)	44	51.8	149	44.9	1.35	0.84 to 2.17	0.218
(No)	41	48.2	183	55.1			
Appropriate complementary feeding for children aged 6–24 months. (yes)	63	74.1	141	42.5	3.88	2.28 to 6.60	<0.001†
(No)	22	28.9	191	57.5			
Good nutrition for women of childbearing age (yes)	76	89.4	138	41.6	11.87	5.75 to 24.50	<0.001†
(No)	9	10.6	194	58.4			
Production and consumption of diverse nutritious foods all year round. (yes)	70	82.4	125	37.7	7.73	4.24 to 14.08	<0.001†
(No)	15	17.6	207	62.3			
Production and consumption of Biofortified crops. (yes)	50	58.8	78	23.5	4.65	2.82 to 7.68	<0.001†
(No)	35	41.2	254	76.5			
Hand washing at the five critical times for all household members (yes)	82	96.5	199	59.9	18.27	5.65 to 59.03	<0.001†
(No)	3	3.5	133	40.1			
Hygiene enabling facilities (yes)	81	95.3	253	76.2	6.32	2.25 to 17.80	<0.001†
(No)	4	4.7	79	23.8			
Hygiene enabling facilities							
Tippy Taps (yes)	60	70.6	78	23.5	7.82	4.60 to 13.29	<0.001†
(No)	25	29.4	254	76.5			
Rubbish pit (yes)	70	82.4	216	65.1	2.51	1.37 to 4.57	0.002†
(No)	15	17.6	116	34.9			
Improved latrine (toilets) (yes)	46	54.1	159	47.9	1.28	0.80 to 2.07	0.306
(No)	39	45.9	173	52.1			
Pot rack (yes)	76	89.4	239	72.0	3.29	1.58 to 6.83	0.001†
(No)	9	10.6	93	28.0			

*Binary logistic regression, p value.

†P value significant at p<0.05. Hygiene enabling facilities=presence of tippy taps, rubbish pits, improved latrines and pot racks.

‡P value from Independent samples t-test.

HDDS, Household Dietary Diversity Score; MDD-W, Minimum Dietary Diversity for Women; WASH, water, sanitation and hygiene.

### Food security and dietary diversity

HDDS and MDD for women (MDD-W) were significantly higher in exposed (96.5%) compared with controls (49.4%) (p=0.005). This finding corresponded with qualitative data recorded from the KIIs and FGDs. For instance, majority of the KII mentioned the increased production of diversified crops (including small grains) as well as small livestock (chicken, fisheries) production to ensure availability of four-star diets. Adoption of production and consumption of biofortified crops especially NUA 45 beans and orange sweet potatoes was also highlighted. The participants from FGDs held agreed that care groups have resulted in knowledge exchange especially on types of crops that can be grown all year round with high nutritive values within the concept of using locally available ‘traditional’ foods.

### Knowledge and practice of promoted behaviours

Our findings showed that knowledge of the seven promoted behaviours was also higher in the exposed compared with controls. The proportion of participants who remembered at least five behaviours was higher in exposed (96%) compared with controls (49.4%) (p<0.001). Key informants strongly recommended that the consortia should promote all the seven behaviours in all participating districts. The FGD participants highlighted that here are inherent socio-cultural and religious barriers to EBF and adoption of recommended IYCF behaviours.

### Hygiene enabling facilities

Hygiene enabling facilities were significantly higher in ZRBF wards with the exception of improved latrines (p=0.306). Similar to the quantitative data there were field observations of pot racks, rubbish pits, tippy taps and advanced latrines in some households. The proportion of exposed ‘practising hand washing at the five critical times for all household members’ was significantly higher compared with controls (p<0.001).

## Discussion

The current study showed that knowledge and practice of the promoted behaviours was generally higher in exposed group compared with the control group with exception of EBF and CDDS. These findings add on to existing evidence that shows the potential of using the CGA as a vehicle for the integration of nutrition within existing community programme to achieve changes in behaviours.^13 24 25^ Nonetheless a multisectoral approach is required to ensure sustainability and scaling up to non-participating districts using lower level governance structures.^7^

### Knowledge and practice of promoted behaviours

In this study, knowledge and practice of all promoted behaviours were significantly higher in the exposed compared with the control group with exception of EBF. The results are similar to earlier findings from Zimbabwe.^10 26^ However, the lack of impact of the CGA on the practice of EBF is not surprising considering that EBF is a behaviour universally promoted in Zimbabwe by the ministry of health in line with UN SDGs agenda 2030.^27^ In 2019, 41.9% of the mothers practised EBF in Zimbabwe.^28^ Furthermore, breastfeeding behaviours are usually promoted in a culturally appropriate manner thus enhancing adoption.^29–31^

### Care groups and improved dietary diversity

Interestingly, HDDS and MDD-W were significantly higher in exposed than controls in the current study. Our findings reflect a positive impact of the CGA on increased household food supply and agree with earlier findings from Cambodia, Mozambique, Malawi, Kenya and Rwanda.^7^ The ZRBF integrated agriculture, nutrition and livelihoods intervention brought significant improvements in consumption of diversified diets possibly via promotion of nutrition gardens, traditional grains and small livestock production. The mainstreaming of nutrition education and social behaviour change initiatives via the CGA is recommend for future programmes.

However, it is concerning that the improved diet quality at the household level is not translating to improved child diet quality. This could be as a result of traditional and cultural beliefs that support poor intrahousehold food distribution that disadvantages children within the African context.^32^ Therefore, future studies that investigate factors affecting intra household food distribution, and at large social determinants of children’s dietary intake are required.

### Care group approach, child illnesses and WASH

Our results showed that there was no significant difference the two groups on the prevalence of diarrhoea, cough and fever. These results contradict positive impact of CGA on child morbidity and mortality reported in studies from Ethiopia,^33^ Cambodia, Mozambique, Malawi, Kenya and Rwanda with decline in child mortality.^7^ While a study in Uganda that assessed the contribution of volunteer community health workers on child morbidity reported a decreased trend.^34^ The lack of significant effect on child morbidities in the current study is surprising considering that hygiene enabling facilities were significantly higher in the participating wards. Similar findings in Zimbabwe were previously reported by Gomora *et al*,^9^ ^10^ and Macheka *et al.* 26 Furthermore, the prevention and management of child morbidities is also influenced by community and national level factors which requires a multifaceted approach.

### Strengths and limitations of the study

Our study had several strengths. We used a retrospective cohort design. The mixed-methods approach used makes the study design and findings more robust. While in a quantitative design the relationships between variables are measurable, qualitative design helps elucidate drivers of human behaviours. Although, diet quality was assessed using validated methodology,^20–22^ the limitations of recall bias should be considered. Retrospective cohort studies may prove an association but do not demonstrate causation. Overall the results should be interpreted with the understanding that counselling ‘care groups’ has limited impact on nutritional status of children.^35^ Lastly, the impact or the lack of it could have been as result of confounding programmes and/or potential contamination that might have occurred in the control wards.

## Conclusions

The results showed that knowledge and practice of all promoted behaviours were significantly higher in the exposed than in controls with exception of EBF. However, there was no significant difference between exposed and controls on the prevalence of; diarrhoea, cough and fever, CDDS. Interestingly, HDDS and MDD-W were significantly higher in exposed than controls. Considering the limited impact of counselling ‘care groups’ on nutritional status of children there is need to its mainstreaming into existing nutrition, agriculture and livelihoods programmes for maximum impact.

## Data Availability

Data are available on reasonable request. The anonymised datasets used and/or analysed during the current study are available from the corresponding author, TMM on reasonable request. Email: tmatsungo@gmail.com.
